# Jugular foramen dural arteriovenous fistula: A case report and literature review

**DOI:** 10.1097/MD.0000000000042836

**Published:** 2025-06-20

**Authors:** Zhangcai Yan, Jingfang Hong, Jialong Ji, Xainqun Wu, Shousen Wang, Haibing Liu

**Affiliations:** a Department of Neurosurgery, Pucheng County General Hospital, Nanping, Fujian, China; b Department of Neurosurgery, 900th Hospital (Fujian Medical University), Fuzhou, China.

**Keywords:** ascending pharyngeal artery, bulbar paralysis, dural arteriovenous fistula, endovascular therapy, jugular foramen

## Abstract

**Rationale:**

Clinically, jugular foramen (JF) dural arteriovenous fistula (DAVF) is rare, and treatment is even more difficult.

**Patient concerns:**

A 67-year-old woman with progressive left eye distension and visual acuity decline.

**Diagnoses:**

A digital subtraction angiography examination revealed a JFDAVF, which showed the feeding artery is ascending pharyngeal artery, with retrograde flow through the inferior petrosal sinus into the ophthalmic vein.

**Interventions:**

An endovascular interventional therapy method was chosen, Marathon microcatheter in synchro-10 Microguide wire auxiliary super selected to ascending pharyngeal artery, about 0.3 mL of 13% concentration GLUBRAN was injected with a Marathon microcatheter, post-embolization angiography confirmed obliteration of the fistula site.

**Outcomes:**

Aspiration when drinking and hoarseness after endovascular embolization, after 3 days, the eye symptoms completely disappeared. after 3 months, no aspiration observed while drinking and normal articulation. the patient recovered well post-embolization.

**Lessons:**

Therefore, endovascular treatment is an appropriate choice for JFDAVF, but, arterial approach is very easy to develop neurological dysfunction.

## 1. Introduction

Dural arteriovenous fistulas (DAVF) are abnormal acquired intracranial vascular malformations consisting of pathological connections located within the dura between the pial arteries and the veno vasora, comprising the walls of the dural sinuses, bridging veins, or transosseous emissary veins.^[1,2]^ Su et al^[3]^ define fistulous points as the locations where the feeding arteries transition from thick to thin and the draining veins transition from thin to thick. Presenting symptoms are variable depending on the location of the fistula and can include pulsatile tinnitus, bruit, headaches, visual changes, alterations in mental status, seizure, myelopathy, cranial nerve palsies, and motor or sensory deficits. Approximately 20% to 33% of DAVF present with intracranial hemorrhage,^[4,5]^ high-grade aggressive lesions can cause hemorrhagic events and nonhemorrhagic neurologic deficits if left untreated.^[6]^ Bulboconjunctival hyperemia and swelling are the most common symptoms of cavernous sinus (CS)-DAVF,^[7]^ However, there was no report of the first ocular symptom of jugular foramen (JF)DAVF.^[8]^ It is believed that this is the first reported case of JFDAVF presenting with ocular symptoms.

## 2. Patient information

**Figure 1.**
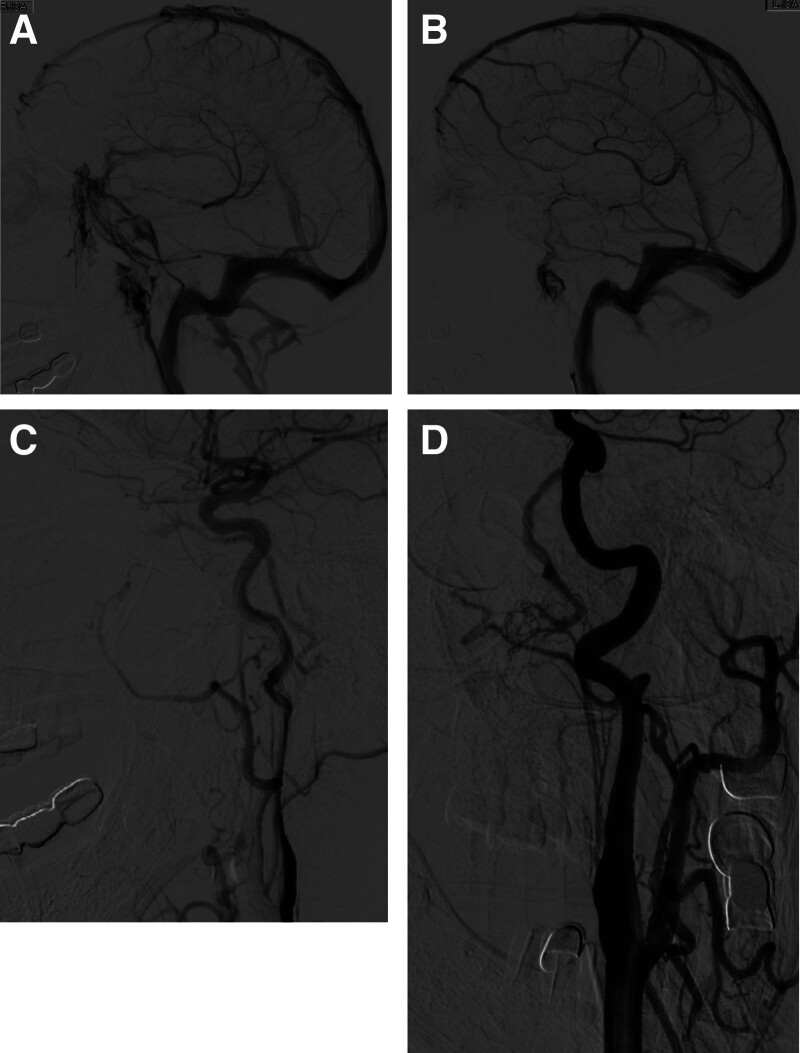
Preoperative head DSA examination. (A) Right cavernous sinus-inferior petrosal sinus forward drainage (arrow). (B) Left inferior petrosal sinus were not visualized (arrow). (C, D) Lateral imaging of the left common carotid artery revealed JFDAVF, which was countercurrent to inferior petrosal sinus-cavernous sinus-superior ocular vein, and drainage was carried out through the facial vein (arrow), APA supply fistula (arrow). APA = ascending pharyngeal artery, DSA = digital subtraction angiography, JF = jugular foramen, DAVF = dural arteriovenous fistula.

Patient Lin XX, female, 67-years-old, had no relevant medical history presented with headache for 20 days, left eye distending pain, vision loss progressive worsening for 12 days. COVID-19 infection 1 month ago. Physical examination: left pupil diameter of 6 mm, direct and indirect light reflection disappeared, visual acuity: right eye 0.5, accurate light positioning, left eye 0.2, accurate light positioning, intraocular pressure: right eye 15 mm Hg, left eye 37 mm Hg. Protruding left eye ball, eyelid swelling, full eye socket, normal skin temperature, no obvious sense of fluctuation, eyelid ptosis, covering the upper two thirds cornea, incomplete eyelid closure, eye fixation, and limited movement in all directions. Conjunctival mixed congestion, edema (+++), the conjunctival vessels are highly tortuous and dilated. There was no abnormality in computed tomographic angiography and computed tomographic venography of the head in other hospitals. Previously diagnosis at another hospitals: Iridocyclitis. Symptoms progressively deteriorate during administration of anti-infective and corticosteroid therapy. Transferred to ophthalmology department from another hospital. ophthalmology department diagnosis: Left Rochon-Duvigneaud syndrome. Consider the possibility of cerebrovascular disease, asked me for a consultation. Considering the possibility of CS-DAVF transfer to Neurosurgery. Digital subtraction angiography (DSA) to confirm the diagnosis of left JFDAVF, the feeding artery is ascending pharyngeal artery (APA), with retrograde flow through the inferior petrosal sinus into the ophthalmic vein, and subsequent drainage via facial vein (Fig. 1A–D). Complete assessment of image data, definitive treatment indications was identified, yet craniotomy was declined by the patient, owing to the anatomical constraints preventing venous access to the fistula, transarterial embolization become the obligatory strategy.

**Figure 2.**
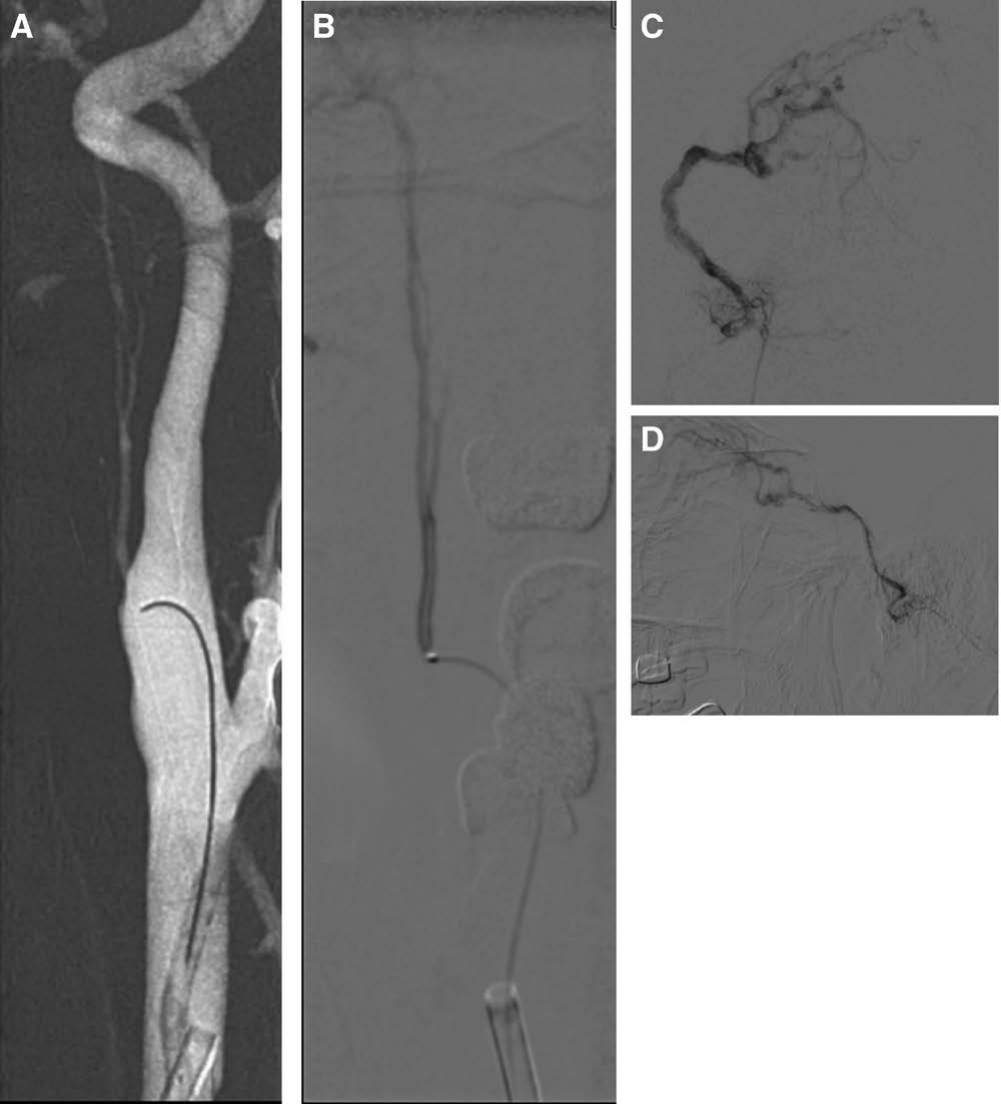
During surgery. (A) Left common carotid artery 3-D imaging, select the working angle of the super selected APA (arrow). (B) After the Marathon microcatheter was inserted into APA, the APA was confirmed to be the blood supplying artery by angiography (arrow). (C, D) The microcatheter is close to fistula, angiography showed multiple small branch blood supply fistulas, which were drained by single vein and countercurrent to inferior petrosal sinus-cavernous sinus-superior ocular vein (arrow). APA = ascending pharyngeal artery.

**Figure 3.**
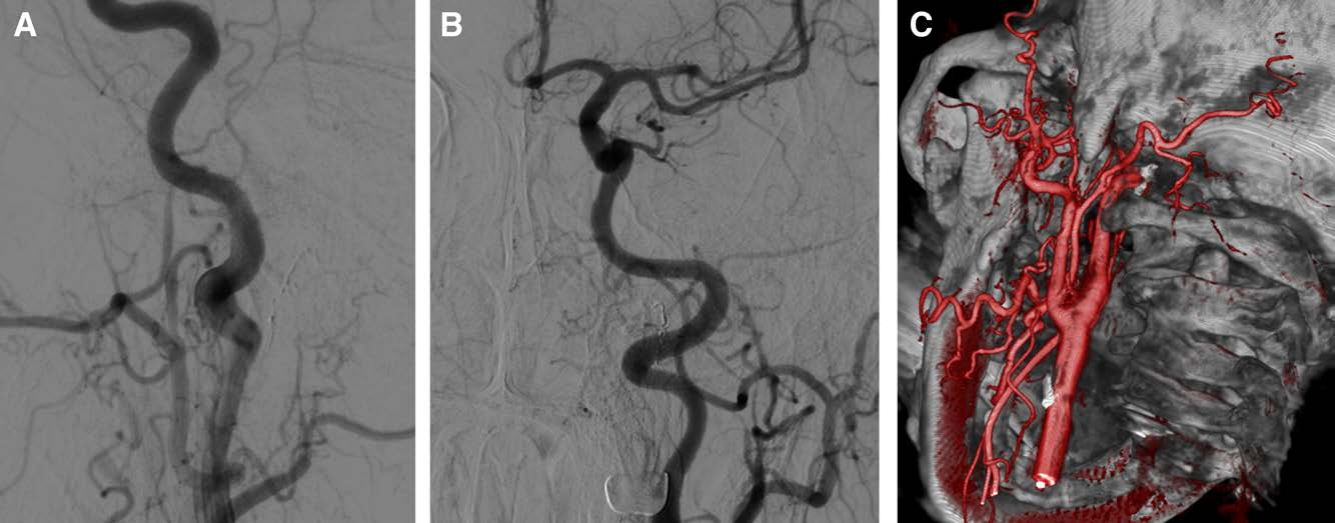
Review after surgery. (A, B) Postoperative, no abnormal venous (arrow). (C) Postoperative Xper-ray CT plain scan and preoperative 3-D reconstruction fusion showed that the glue was located in the jugular foramen (arrow).

The 6F guiding envoy (Codman) was enter into the initiation of the left internal carotid artery with the aid of a guide wire, Marathon microcatheter (Micro Therapeutics Inc. Dba ev3 Neurovascula) in synchro-10 (Stryker Neurovascular) Microguide wire auxiliary super selected to initial part of APA, microcatheter hand-injection angiography further confirmed APA as the feeding vessel, demonstrating direct communication with the fistula (Fig. 2B), the microcatheter angiograph near the fistula showed multiple small branches feeding the fistula (Fig. 2C, D), approximately 0.3 mL of 13% concentration GLUBRAN (Micro Therapeutics Inc. dba ev3 Neurovascula) was injected through the Marathon microcatheter, post-embolization angiography confirmed obliteration of the fistula site (Fig. 3A, B). Post-embolization Xper-CT and pre-embolization 3-D of DSA image fusion were performed, Glue is located on the left JF (Fig. 3C). Aspiration when drinking and hoarseness after endovascular embolization. After 3 days, the eye symptoms completely disappeared. After 3 months, no aspiration observed while drinking and normal articulation. Six months after discharge, magnetic resonance angiography and magnetic resonance venography of the head showed no abnormal blood vessels. One year after discharge, DSA revealed no abnormal vascular in the left JF.

## 3. Discussion

### 3.1. Characteristics of this case

One month ago, the patient was infected with COVID-19, 20 days ago, he developed headache, left orbital swelling, bulbar conjunctival hyperemia, and progressive vision decline. Whether to have cerebral venous thrombosis, cause vein circumfluence obstacle, corresponding induced headache, because of the lack of relevant inspection, was unable to confirm thrombus. However, the inferior petrosal sinus was not developed in the ipsilateral internal carotid angiography. However, the venous drainage of the fistula was retrograde through the inferior petrosal sinus, cavernous sinus, and superior ophthalmic vein. Therefore, it is reasonable to believe that there is positive blood flow in the original inferior petrosal sinus. It could be caused by a thrombosis caused by infection with COVID-19.^[9,10]^

### 3.2. Anatomy of the jugular foramen

The JF is divided into 3 compartments: 2 venous and a neural or intrajugular compartment. The use of a partition can effectively cater to the requirements of microsurgery. The glossopharyngeal, vagus, and accessory nerves penetrate the dura on the medial margin of the intrajugular process of the temporal bone to reach the medial wall of the internal jugular vein. The first branch of the APA, often provides the most prominent supply to the meninges around the JF.^[11]^ The APA is a small, but important artery that supplies multiple cranial nerves and anastomotic channels to the anterior and posterior cerebral circulations. To evaluate and treat such diseases, it is necessary not only to know selective angiography and embolization techniques, but also the territory of the APA, anastomoses, and vascular supply to the vasa nervorum of lower cranial nerves.^[12]^ Although the APA often originates from the posterior medial wall of the external carotid artery, there are sporadic reports of common carotid artery bifurcation and internal carotid artery origin.^[13,14]^ In this case, the APA originated in front of the C1 segment of the internal carotid artery. The APA rises along the lateral wall of the pharynx to the skull base, and the terminal branches of the APA are many and important.

### 3.3. Treatment selection

Treatment options vary based on angioarchitecture, location, and patient characteristics and range from conservative observation to palliative treatment, radiosurgery, endovascular embolization, and craniotomy. The main goal of treatment is to obliterate flow through the abnormal connection and prevent further arterial flow to the venous system.

We searched the PubMed database using the keywords “dural arteriovenous fistula” and “jugular foramen.” We found 5 articles describing DAVF located at the JF and 8 cases are reported.^[8,15–18]^ All published clinical cases were written in English. The clinical information of these cases is summarized in Table 1.

**Table 1:** Review of clinical characteristics of reported cases of jugular foramen dural arteriovenous fistula.

Case no.	Age/sex	Presentation	CC	Feeding artery	Arterial approaches	Materials	Drainage vein	Angiography result	Nerve symptoms	Outcome (mRS)
1	59/M	Tinnitus	IIa	PMA, MMA	MMA	Onyx 1.8 mL, surgery	IJV	Complete	/	0
2	69/M	Hemorrhage	IIb	APA, OA, PAA	APA	Onyx 2 mL	Cortical veins	Complete	/	0
3	47/F	Tinnitus	I	MMA, PAA, PMA	MMA	Onyx 1 mL	IJV	Complete	/	0
4	28/M	Tinnitus	I	MMA, PAA, PMA	PAA	Onyx 2.5 mL	IJV	Complete	/	0
5	52/F	Bilateral lower extremity weakness and incontinence of urine and feces		AphA, OA	OA	Onyx 0.6 mL	Anterior and posterior spinal veins	Complete	/	0
6	64/M	Subarachnoid and intraventricular hemorrhage		The neuromeningeal trunk of the right APA	APA	25% glue, 0.6 mL	The right lateral medullary veins craniopetally	Complete	Bulbar paralysis	2
7	81/M	Headaches and left homonymous hemianopia, right temporal intracerebral hematoma	III	OA, and branches from the posterior auricular and APA	Microsurgical	/	Cortical venous	Complete	Mild residual hemianopia	1
8	70/M	Right-sided medullary hemorrhage with pronounced Wallenberg syndrome.	IV	Multiple branches of the ECA and VA	Transarterial embolization, microsurgical	Onyx 34	The ascending pontomesencephalic vein	Complete	/	0

APA = ascending pharyngeal artery, CC = cognard classification, F = female, GOS = Glasgow Outcome Scale, IJV = internal jugular vein, M = male, MMA = middle meningeal artery, OA = occipital artery, PAA = posterior auricular artery, PMA = posterior meningeal artery, VA = vertebral artery.

In the course of endovascular therapy, the vessel should be confirmed by microcatheter angiography, and the appropriate microcatheter and microguide wire should be selected to avoid the vascular rupture caused by the microcatheter branching.

During endovascular treatment, microguide angiography should be used to confirm the origin of the artery and the position of the main artery, and the appropriate microcatheter and microguide wire should be selected to avoid the microcatheter stray into the branches and cause vascular rupture.

In 1988, therapeutic embolization of an APA-internal jugular vein fistula, 5 tiny pieces of Gelfoam and 20 particles of Ivalon (250–590 μ in size), all soaked in water-soluble contrast medium, were injected under fluoroscopic control, 3 years later the patient remains asymptomatic apart from the persistence of a left sensorineural hearing loss as shown by a follow-up audiometric study.^[19]^ Pero performed injections of Onyx in the superior pharyngeal branch of the APA in 3 cases to occlude aggressive CS-DAVFs that were not reachable by more common venous catheterization routes.^[20]^ Fang treated 13 cases of skull base DAVF by APA, although 2 patients who were treated with APA had complications (1 patient had glue leakage and 1 had APA rupture), no neurologic symptoms were present during the postoperative period and follow-up.^[21]^ It can be seen from the article that APA compensatory augmentation is obvious, blood flow is large, and the steal phenomena leads to APA not supplying blood to nerves.^[22]^ Cranial nerve palsy is also a considerable issue during embolization via the NMT of the APA. Catheterization of the superior pharyngeal branch of the APA is safe because it is completely extracranial and far away from the origin of the neuromeningeal branch of the APA, lowering the risk of occlusion of this branch due to Onyx reflux.^[13]^

Meticulous analysis of the angioarchitectural characteristics and clinical implications is warranted for safe and effective treatment.^[23]^ The use of an individually tailored transcondylar approach to treat DAVF at the region of the JF is most effective. This approach allows for complete obliteration of the connecting arterial feeders, and removal of bony structures containing pathological vessels.^[24]^ There were no additional neurological deficits, with the exception of patient 4, who experienced mild hypoglossal nerve palsy postoperatively.

Symptomatic sinus occlusion complicated with DAVF, balloon angioplasty plus mechanical cracking (pulling a microcatheter back and forth) was used to recanalize the occluded sinuses, the symptoms disappeared after endovascular recanalization of the occluded left transverse sinus and sigmoid sinuses, and follow-up venography revealed opened sinuses with complete disappearance of the DAVF.^[25]^ The imaging data of this patient were retrospectively analyzed, recanalization of the occluded inferior petrosal sinus and let the vein drain forward, it may be a better choice.

To conclude, JFDAVF uncommonly rare and difficult to treat, and they therefore pose substantial challenges during diagnosis and management. DSA is the most critical objective basis for treatment choice. If there is no good venous access, arterial approach may be cured, but it is very easy to develop neurological dysfunction, surgery may also be a good option, in the future, recanalization of venous sinus occlusion may also be a treatment for DAVF related to venous sinus occlusion.

## Acknowledgments

The authors wish to thank the patient whose case is reported.

## Author contributions

**Conceptualization:** Zhangcai Yan, Jingfang Hong, Jialong Ji, Xainqun Wu, Shousen Wang, Haibin Liu.

**Data curation:** Zhangcai Yan, Jingfang Hong, Jialong Ji, Haibin Liu.

**Formal analysis:** Zhangcai Yan, Jialong Ji, Haibin Liu.

**Funding acquisition:** Haibin Liu.

**Investigation:** Haibin Liu.

**Methodology:** Haibin Liu.

**Project administration:** Haibin Liu.

**Resources:** Haibin Liu.

**Software:** Haibin Liu.

**Supervision:** Haibin Liu.

**Validation:** Haibin Liu.

**Visualization:** Haibin Liu.

**Writing—original draft:** Zhangcai Yan, Haibin Liu.

**Writing—review & editing:** Zhangcai Yan, Jingfang Hong, Jialong Ji, Xainqun Wu, Shousen Wang, Haibin Liu.
